# Legacy effects of herbivory on treeline dynamics along an elevational gradient

**DOI:** 10.1007/s00442-022-05125-8

**Published:** 2022-02-11

**Authors:** Ida M. Mienna, Gunnar Austrheim, Kari Klanderud, Ole Martin Bollandsås, James D. M. Speed

**Affiliations:** 1 Faculty of Environmental Sciences and Natural Resource Management, Norwegian University of Life Sciences, Box 5003, 1432 Ås, Norway; 2 Department of Natural History, NTNU University Museum, Norwegian University of Science and Technology, 7491 Trondheim, Norway

**Keywords:** Birch, Dendrochronology, Global warming, Forest expansion, Herbivore density experiment, Sheep grazing

## Abstract

Treelines are expected to expand into alpine ecosystems with global warming, but herbivory may delay this expansion. This study quantifies long-term effects of temporally varying sheep densities on birch recruitment and growth in the treeline ecotone. We examined treeline ecotone successional trajectories and legacy effects in a replicated experimental setup, where enclosures were present for 14 years with three different sheep densities (0, 25, 80 sheep km^−2^). Before and after the enclosures were present, the site had an ambient sheep density of 20–25 km^−2^. We sampled field data 4 years after enclosure removal and compared these to data sampled 8 and 9 years after enclosure erection. We sampled data on birch browsing pressure, birch distribution across life-stages (recruits, saplings, and mature trees), and birch annual radial growth. Fourteen years of increased or decreased sheep density had observable legacy effects depending on birch life-stage. Birch recruit prevalence decreased in areas, where sheep were reintroduced after being absent for 14 years. For the same areas, sapling and mature tree prevalence increased, indicating that these areas have entered alternative successional trajectories compared to areas, where sheep were present the whole time. Birch annual radial growth showed a lag effect of 2 years after enclosure removal, with growth decreasing in areas where sheep had been absent for 14 years and increasing where sheep densities were high. Thus, decadal-scale absences of herbivores can leave legacy effects due to increased numbers of trees that have high resistance to later-introduced herbivore browsing.

**Supplementary Information:**

The online version contains supplementary material available at 10.1007/s00442-022-05125-8.

## Introduction

The treeline ecotone, defined as the transition zone between forests and treeless areas including the upper distribution limit of mature trees, is expected to expand into higher elevations and latitudes due to global warming (Kaplan and New 2006). This will reduce the extent of alpine ecosystems. Temperature is thought to be the main driver of treeline elevations (Paulsen and Körner 2014). However, a meta-analysis of 166 treeline sites across the globe found that less than half of the treelines had increased in elevation with climate warming (Harsch et al. 2009). This suggests that other drivers, such as human land use and herbivory, may also limit treelines (Cairns and Moen 2004; Tømmervik et al. 2004; Gehrig‐Fasel et al. 2007; Speed et al. 2010; Bello-Rodriguez et al. 2019). Browsing by herbivores may affect processes, such as tree recruitment and growth, and ultimately the successional speed and trajectory of an ecosystem. High densities of herbivores can inhibit expansion of woody vegetation by reducing the growth and survival of trees (Hobbs 1996; Cote et al. 2004). For areas with no herbivores, tree recruitment can be high (Speed et al. 2010; Frei et al. 2018). Thus, changes in herbivore densities can lead to alternative successional trajectories, which over time can lead to a change in ecosystem state (Suding et al. 2004; Cuddington 2011; Hidding et al. 2013). The new ecosystem state can then persist a long time after herbivore browsing is removed, partly due to legacy effects (Cuddington 2011). In ecosystems where woody vegetation is sparse due to colder temperatures, such as in alpine and arctic areas, a combination of climate warming and removal of persistent browsing pressure may lead the system towards an alternative successional trajectory, which over time might lead to a stable forest ecosystem state. For alpine ecosystems, herbivory can increase plant community recovery rates after environmental changes, and thus inhibit the system from entering a successional trajectory towards forest (Olsen and Klanderud 2014). For woody-dominated ecosystems, such as forests, alterations in herbivore densities can shift the successional trajectory of the forest from high to less palatable vegetation (Hidding et al. 2013). Thus, herbivory seems to affect woody and non-woody ecosystems differently.

Long-term human land use strongly impacts treeline dynamics due to livestock grazing, fuelwood cutting, and haymaking. Hence, current treelines, particularly in Europe, are often legacies of historical land use (Staland et al. 2011; Schworer et al. 2015; Wielgolaski et al. 2017). Livestock densities and the intensity of human land use have been decreasing in several mountainous areas across Europe in the last decades (MacDonald et al. 2000), leading to lower overall browsing pressure from livestock which may cause upward shifts in treeline elevation. Speed et al. (2010, 2011b) found that after 8 years, enclosures spanning the treeline ecotone without the presence of sheep had higher recruitment of trees and higher tree-ring growth compared to enclosures with sheep present. Browsing also overrode the influence of temperature in determining birch tree-ring growth (Speed et al. 2011b). Thus, changes in browsing by herbivores may determine the successional trajectory of treeline ecotones. However, to study the effect of herbivory on treeline ecotone successional trajectories and test whether temporal variations in herbivore densities can leave legacy effects, studies of treeline dynamics across fluctuating herbivore densities are required.

**Fig. 1:**
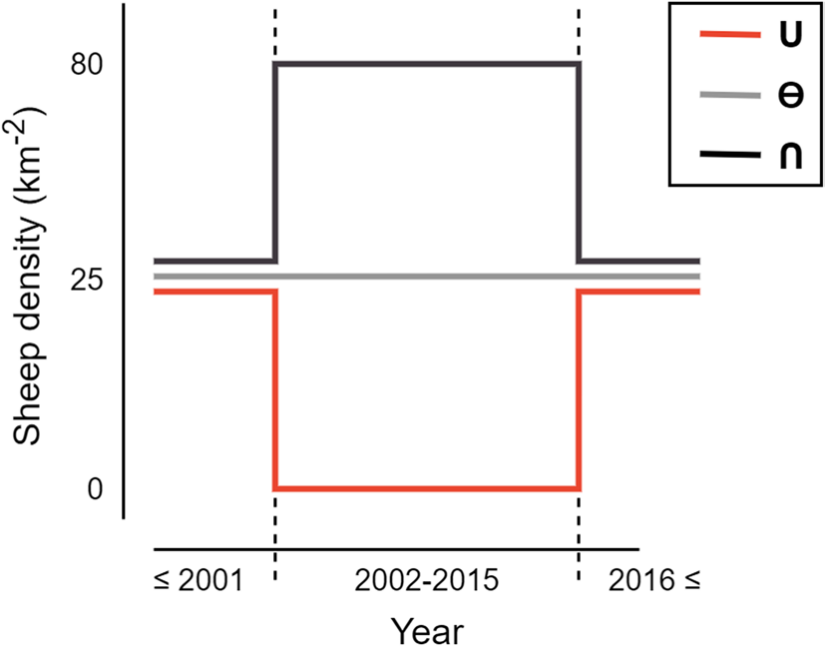
Conceptual figure of the three treatments before the enclosures were erected (≤ 2001), when the enclosures were present (2002–2015) and after the enclosures were removed (2016 ≤). The **∪** treatment had no sheep within the enclosures and a low sheep density before 2002 and after 2015. The **Ө** treatment had an overall low sheep density (25 sheep km^−2^) before, during and after the enclosures were present. The **∩** treatment had a high sheep density (80 sheep km^−2^) within the enclosures and a low sheep density before 2002 and after 2015. The dashed lines represent the erection and removal of enclosures in 2002 and 2015, respectively. The sheep density numbers are approximate

In this study, we examine treeline ecotone successional trajectories and legacy effects using an experimental setup along an elevational gradient with varying herbivore densities over two periods in time. Between 2002 and 2015, the study area, an elevational gradient in the mountains of Norway, was divided into three experimentally different sheep densities (0, 25 and 80 sheep km^−2^). Results from this period on how herbivory affects the prevalence and growth of mountain birch (*Betula pubescens* subsp. *Czerepanovii*) in the treeline ecotone are reported in Speed et al. (2010, 2011a, b). Prior to 2002 and after 2015 the whole site had an ambient sheep density of around 20 to 25 sheep km^−2^. The different combinations of sheep densities in our study are thus: (1) a decrease followed by a return to ambient sheep density (represented by the symbol **∪**), (2) continued ambient sheep density (represented as **Ө**), and (3) an increase followed by a return to ambient sheep density (represented as **∩**) (Fig. 1). We analyse how changes in sheep densities affected (1) browsing pressure on birch, (2) prevalence of birch trees in different life-stages (recruits, saplings and mature trees), and (3) birch radial growth along an elevational gradient in the treeline ecotone. By comparing treeline birch data sampled 8 and 9 years after the initiation of the sheep density treatments (2009 and 2010) and 4 years after the termination (2019), we analyse if the herbivore experiment resulted in alternative successional trajectories.

We have different predictions (P1–P5) of legacy effects across treatments (Table 1). (P1) For the **Ө** treatment, we predict no changes in browsing pressure, birch prevalence or birch radial growth between 2009 and 2019 because of the continued ambient sheep density. (P2) We expect increased browsing pressure with the increased sheep density after 2015 in the **∪** treatment, and as the probability of being browsed is highest for recruits and saplings (< 175 cm; Speed et al. 2011a), we predict fewer recruits and saplings in 2019 compared to 2009 after reintroducing sheep in the **∪** treatment. Thus, we expect no legacy effects for birch in these life-stages in this treatment. (P3) On the other hand, because of expected decreased browsing pressure from 2009 to 2019 in the **∩** treatment, we predict to find more recently recruited trees in this treatment in 2019 compared to 2009. (P4) We predict more mature trees (height ≥ 175 cm) in 2019 than in 2009 in the **∪** treatment, as these would have escaped the increased browsing by reintroduced sheep. More mature trees can indicate that the treeline ecotone has entered a successional trajectory towards a forest ecosystem. For the **∩** treatment we do not expect an increased prevalence of mature trees as this birch life-stage did not vary between the **∩** and **Ө** treatment in 2009. (P5) Following previous findings on birch radial growth as a response to sheep density (Speed et al. 2011b), we predict a decrease in tree-ring increment for the **∪** treatment and an increase in tree-ring increment for the **∩** treatment.

**Table 1:** Previous findings and our predictions based upon previous findings

Aim		Findings after erection of enclosures^a^			Predictions after removal of enclosures		
		**∪**	**Ө**	**∩**	**∪**	**Ө**	**∩**
1	Browsing pressure	–	0	+	+ (P2)	0 (P1)	– (P3)
2	Recruit prevalence	+	0	–	– (P2)	0 (P1)	+ (P3)
2	Sapling prevalence	0	0	0	– (P2)	0 (P1)	+ (P3)
2	Mature trees prevalence	0	0	0	+ (P4)	0 (P1)	0 (P4)
3	Radial growth	+	0	–	– (P5)	0 (P1)	+ (P5)

Previous findings by Speed et al. (2010, 2011a, b) are either positive (+), negative (–) or not different (0) compared to findings in the ambient sheep density (**Ө**). Our predictions are compared to findings by Speed et al. (2010, 2011a, b) for the same treatment. Specific predictions after removal of enclosures P1–P5 are included in brackets for each combination of treatment and response

^a^Results from Speed et al. (2010, 2011a, b)

## Materials and methods

### Study area

The study was conducted at a site in the treeline ecotone in Hol municipality in southern Norway (60° 40′–60° 45′ N and 7° 55′–8° 00′ E). The experimental site is south facing and ranges in elevation from 1050 to 1320 m a.s.l. The vegetation in the area mainly consists of dwarf shrub heathlands, but also lichen-dominated ridges and graminoid-dominated meadows (more details in Austrheim et al. 2005). Mountain birch (*Betula pubescens* subsp. *czerepanovii*) is the treeline forming species at the site, but also other tree species, such as Norway spruce (*Picea abies*), Scots pine (*Pinus sylvestris*), rowan (*Sorbus aucuparia*) and aspen (*Populus tremula*), are present. Other ungulates such as wild reindeer (*Rangifer tarandus*) and moose (*Alces alces*) visit the area only occasionally. Mountain hare *(Lepus timidus*) is also present, but we found no signs of hare browsing that could have affected the results of the current study. There were also no signs of birch moth outbreaks in the area between 2002 and 2019. Climate data for the study area are represented by interpolations between the official weather stations closest to the site (Norwegian Meteorological Institute; Tveito et al. 2005). The mean temperature ranges from − 8.5 °C during the winter months (December, January, February) to 8.4 °C during the summer months (June, July, August) and mean annual precipitation of 729 mm.

**Fig. 2:**
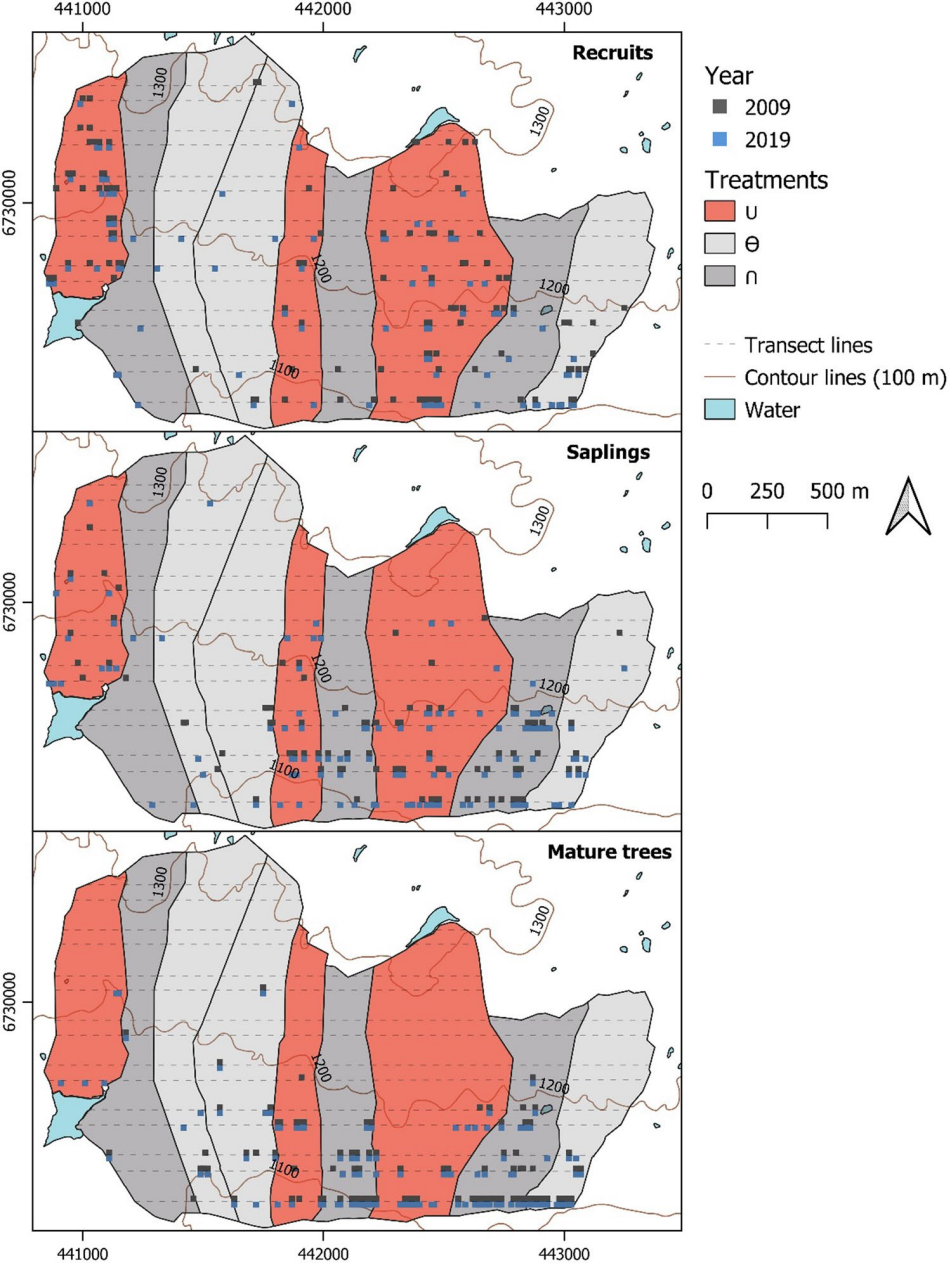
Prevalence of birch trees in the three life-stage classes: recruits (height < 175 cm and basal stem diameter ≤ 15 mm), saplings (height < 175 cm and basal stem diameter > 15 mm), and mature trees (height ≥ 175 cm) in 2009 and 2019 for the three different treatments: a decrease followed by a return to ambient sheep density (**∪**), continued ambient density (**Ө**), and an increase followed by a return to ambient sheep density (**∩**). Squares represent presence of at least one birch belonging to a certain life-stage class within 10 × 10 m segments of transects. The squares are nudged along the *y*-axis so that they do not overlap. Dashed horizontal lines represent the transect lines used for surveying for birch prevalence. Map projection: WGS84/UTM zone 32 N

Between 2002 and 2015, nine enclosures were present at the site (Fig. 2), each covering an average area of 0.3 km^2^ (Austrheim et al. 2008). The enclosures were established along the slope and grouped into three blocks that each had three sheep density treatments: no sheep, low sheep density (25 km^**−2**^) and high sheep density (80 km^**−2**^). Treatment for each enclosure was randomly assigned within each block. Sheep were present in the low- and high-density enclosures between late June to early September, which is the normal summer grazing practice in the area. Before the enclosures were erected in 2002 and after they were removed in 2015, the site was estimated to have an ambient sheep density of around 20–25 sheep km^**−2**^. Each treatment is henceforth presented by symbols representing the change in sheep density from before, during and after the enclosures were present: the no sheep treatment had a decrease when enclosures were erected and then a return to the ambient sheep density when enclosures were removed (**∪**), the low sheep density treatment had no change in sheep density during the whole period and thus had an ambient sheep density (**Ө**), and the high sheep density treatment had an increase followed by a return to ambient sheep density (**∩**) (Fig. 1).

### Data sampling of browsing pressure, birch prevalence and radial growth

To assess the effect of change in sheep density on the treeline ecotone, we sampled data on browsing pressure, birch prevalence and annual radial growth following the same procedures as in Speed et al. (2010, 2011a, b). The data from the study area was sampled along 23 transects by Speed et al. (2010) running from east to west across the treatments. The transects were evenly distributed with an inter-distance of 60 m, and transect endpoint coordinates were registered using a handheld GPS receiver. In the current study, birch stem discs were sampled in the eight transects that previously had been used for destructive sampling of trees in 2010 by Speed et al. (2011b). Browsing pressure and birch prevalence was recorded in the remaining 15 transects.

For the recording of birch browsing pressure and birch prevalence at different life-stages, we surveyed each of the 15 transects in early July 2019 using a handheld GPS for navigation. To reduce sampling errors, we surveyed the transects in a stratified random order to ensure that there was no temporal bias in the sampling of the elevational gradient. We recorded trees within 10 m wide strips centred along each transect. To later classify the birch trees into different life-stages, we measured tree height from stem base to highest point of the tree, and basal stem diameter using a digital calliper. If a tree had multiple stems (polycormic individual), we measured the largest basal stem diameter. We registered browsing pressure as the proportion of the top 30 shoots that had been browsed for trees with height < 175 cm and basal stem diameter < 30 mm. We recorded coordinates for the largest basal stem using a handheld GPS receiver. Data from 2009 was sampled in the same way as described above.

To measure birch radial growth using tree-rings, we sampled stem discs in mid-July and late September 2019. We searched for trees within 30 m wide strips centred along each transect. We only sampled trees shorter than 2 m in height and < 30 mm in basal stem diameter, as we assumed trees of this size to be affected by sheep, since the enclosures were erected in 2002. To reduce spatial dependency, sampled trees were a minimum of 10 m apart. The sampled trees were mostly upright monocormic, although a few trees had multiple stems. In such cases, we sampled the largest stem. Before sampling polycormic trees, we assessed the trees to ensure that stem discs had not been sampled in the past. We sampled the discs at the base of the stem by cutting off the whole stem, and recorded coordinates, height, and basal stem diameter for each tree.

### Acquisition of enclosure polygon and elevational data

To enable spatial co-registration of the individual tree measurements to treatments, we manually drew polygons for each enclosure using aerial images acquired by the Norwegian mapping authority from before the enclosures were removed. The root-mean-square error for the aerial photography was 0.47 and 0.90 m in longitude and latitude, respectively. We extracted elevation data for the area and for each birch tree from a digital terrain model (DTM) produced from airborne laser scanning (ALS) in 2018 by the Norwegian mapping authority. The ALS data had a mean point density of 2 p m^−2^. The xy-coordinate accuracy of an ALS-point is approximately 20 cm and slightly worse for the z-coordinate (Reutebuch et al. 2003). We assigned trees to enclosures based on their recorded coordinates. For the birch prevalence data, three trees were outside the manually drawn enclosures in 2019 and, therefore, excluded.

### Data processing and analyses

#### Browsing pressure and birch prevalence

To evaluate change in browsing pressure and birch prevalence before and after the enclosures were removed, we compared data recorded in 2009 and 2019. In 2009, all 23 transects were surveyed for trees as described in Speed et al. (2010). However, the comparison of change in browsing pressure and birch prevalence between 2009 and 2019, was based on the 15 transects that were surveyed in 2019 as the remaining eight had previously been used for destructive sampling (Speed et al. 2011b). To examine change in browsing pressure, we used two different browsing pressure responses: browsing likelihood and browsing intensity. We measured browsing likelihood as presence–absence of browsed shoots for each tree and browsing intensity as the proportion of the top 30 shoots that were browsed. For trees with fewer than 30 shoots, we used the total proportion of browsed shoots. We fitted statistical global models for each response against treatment, elevation, year and interaction terms between all variables. We modelled browsing likelihood using logistic regression. We arcsine-transformed browsing intensity and modelled this response using linear regression. To investigate the influence of the different variables on the two browsing pressure responses, we generated a set of sub-models from the global model using the ‘dredge’ function in the *MuMIn* package (Barton 2020) and investigated the top-ranked models based upon conditional AIC (AICc) (Supplementary Tables 1–2). We defined the threshold for a model being considered top-ranked by ΔAICc < 2. For the top-ranked models (Supplementary Table 3), we performed analyses of deviance and covariance, and performed model diagnostics in addition to estimating the model’s goodness-of-fit. For the final logistic and linear regression models, we calculated pseudo *R*^2^ (= 1 − (residual deviance/null deviance)) and adjusted *R*^2^ as a measure of goodness-of-fit, respectively.

To evaluate birch prevalence change between 2009 and 2019, we attributed each observed tree to one of three life-stage classes according to their size: recruits (height < 175 cm and basal stem diameter ≤ 15 mm), saplings (height < 175 cm and basal stem diameter > 15 mm), and mature trees (height ≥ 175 cm). The basal stem diameter threshold of 15 mm to separate recruits from saplings followed Speed et al. (2010) based on how large a tree could be in 2009 to have been recruited after the enclosures were erected in 2002. Mature trees in Scandinavian treeline ecotones are often defined as trees ≥ 200 cm in height (Kullman 1979; Dalen and Hofgaard 2005; Speed et al. 2010; Mienna et al. 2020;). However, in this study, mature trees are represented as trees tall enough to escape browsing by sheep, which was found by Speed et al. (2011a) to be ≥ 175 cm height.

To evaluate change in birch prevalence of the three life-stages between the years, we first divided each transect into 10 × 10 m segments. Second, we presence–absence transformed segments based upon the presence or absence of the three life-stages in 2009 and 2019, thus ending up with six birch prevalence data sets in total. Third, we estimated detection probabilities for the recruit and sapling data sets using the R package *Distance* (Miller et al. 2019; R Core Team 2020) as we assumed detection of birch < 175 cm to vary with year and treatment. To estimate the average detection probability, we fitted models with the half normal and hazard-rate key functions using cosine, Hermite polynomial and simple polynomial adjustments. We selected models for each life-stage class, year and treatment combination based on the minimum Akaike information criterion (AIC) value. We assumed all mature trees were detected during the field survey and thus had a detection probability of 1. Fourth, we modelled all birch prevalence data sets together in one model using logistic regression with year, life-stage, treatment, elevation and interaction terms between each variable as explanatory variables. We added the estimated detection probability as a model offset (Supplementary Table 4). Using the same method as for the browsing pressure models, we generated a set of sub-models (Supplementary Table 5) and investigated the parameter estimates for the top-ranked models (Supplementary Table 6). For the final models, we performed analyses of deviance, model diagnostics and calculated pseudo *R*^2^*.*

#### Birch radial growth

To study the inter-annual effect of sheep densities on tree radial growth, we performed dendrochronological analyses following the methods described in Speed et al. (2011b). We used one stem disc per tree to measure tree-ring increment. Before measuring the tree-rings, we smoothed the upper surface of each disc using a Leitz 1320 microtome and Leitz 1703 Kryomat (Ernst LeitzWetzlar GmbH, Wetzlar, Germany), and we used zinc cream to enhance the contrast between early and late wood. We photographed each disc with a Leica MS5 microscope, Leica DFC 320 R2 digital camera and Leica application suite software v4.0.0 (Leica Microsystems, Wetzlar, Germany). Using ImageJ (Schneider et al. 2012) and the ObjectJ plugin (https://sils.fnwi.uva.nl/bcb/objectj/index.html), we measured tree-rings from pith to bark for four radii at 90°. In total, we measured 79 trees sampled in 2019 to produce mean chronologies with *n* = 40, 20 and 19 for the **∪, Ө** and **∩** treatments, respectively.

We assessed the quality of the tree-ring data by performing correlation analyses and visual inspections of tree-rings within treatments. If these quality assessments indicated potential errors in the tree-ring measurements (e.g., low correlation between rings within same year and treatment), we re-measured the stems to ensure consistency in the measurements. Prior to the analyses, we assumed the ring closest to the bark represented the year 2019, and this ring was discarded as it did not represent the entire growing season. Using the R package *dplR* (Bunn et al. 2020), we calculated basal area increment (BAI) per year from the tree-ring width data, taking into account the growth of previous years (Biondi and Qeadan 2008). The reasoning for using BAI is that tree-ring increment is better represented by tree-ring area than by linear tree-ring width (Biondi and Qeadan 2008; Speed et al. 2011b). Before finalising the dendrochronology, the BAI series was standardised due to biological age trends with tree-ring area decreasing with tree-ring age. The appropriate standardisation method, where the relationship between BAI and tree-ring age was linear, was found to be log_e_(BAI) = *a* + *b* × log_e_(age). Before the standardisation, we excluded the two innermost tree-rings (i.e., the first 2 years of growth) per tree as these created a bias in the standardisation due to high leverage. We back-transformed the residuals from the BAI standardisation and split them into three data sets representing standardised BAI (henceforth labelled BAI_st_) per year for the three treatments.

To test if changes in herbivore densities influenced BAI_st_, we fitted treatment-specific linear mixed-effect models by maximum likelihood with year, elevation and mean summer temperature between the years 1988–2018, in addition to interaction terms between all, as fixed effects and tree ID as random effect. We chose to log-transform BAI_st_ as this gave a better goodness-of-fit. Due to temporal autocorrelation between tree-ring measurements between years, we also added an autoregressive correlation structure (AR1) to account for this. To represent the transitions between enclosures being erected (2002) and removed (2016), we used two time series of data: one sampled in 2010 (Speed et al., 2011b) and the other sampled for this study in 2019. As the data sets represent trees from different years, we made a total of six models: one for each treatment for each data series. We assessed all combinations of predictors using the function ‘dredge’ from the *MuMIn* package (Barton 2020) and investigated all top-ranked models (ΔAICc < 2) (Supplementary Tables 8–10). We refitted the top-ranked models with restricted maximum likelihood to get unbiased parameter estimates. Furthermore, we performed segmented regression using the selected combinations of explanatory variables as model predictors to test if there were changes in slope (i.e., breakpoints over time) for BAI_st_ for each treatment around the years the enclosures were erected and taken down using the R package *segmented* (Muggeo 2008). We used Chi squared likelihood ratio tests to evaluate whether the segmentation improved the model fit (i.e., if the segmented models were significantly better than the non-segmented models).

## Results

### Browsing pressure

Browsing treatment had a significant effect on browsing likelihood and browsing intensity (Table 2).

**Table 2:** Analysis of deviance and analysis of covariance for the first-ranked logistic regression model for browsing likelihood and linear regression model for browsing intensity, respectively

Predictors	Browsing likelihood			Browsing intensity	
	*Df*	Wald Chisq	*p*	*F*	*p*
Year	1	0.01	0.93	1.23	0.27
Treatment	2	80.15	< 0.01	54.92	< 0.01
Elevation	1	0.15	0.70	0.14	0.70
Year × treatment	2	11.93	< 0.01	43.15	< 0.01
Year × elevation	1	7.33	< 0.01	8.78	< 0.01
Treatment × elevation	2	0.57	0.75		
Year × treatment × elevation	2	3.86	0.15		

Level of significance: *p* ≤ 0.05

In the treatment with an overall ambient sheep density (**Ө** treatment), there was no change in either browsing likelihood or intensity between 2009 and 2019 (Supplementary Table 3). For the treatment, where sheep had been absent and then reintroduced (**∪** treatment), browsing likelihood and intensity was lower than in the **Ө** treatment in both 2009 and 2019 (Supplementary Table 3, Supplementary Fig. 1). There was also no significant change in browsing likelihood and intensity between the years for this treatment (Wilcoxon rank sum test: *W* = 77,144, *p* = 0.268 and *W* = 75,782, *p* = 0.080, respectively). For the treatment, where there previously was a high sheep density (**∩** treatment), browsing likelihood did not vary from the **Ө** treatment in neither 2009 nor 2019 (Supplementary Table 3, Supplementary Fig. 1). However, browsing intensity was significantly higher in the **∩** treatment than in the **Ө** treatment in 2009 (Supplementary Table 3, Supplementary Fig. 1). This difference between the two treatments was absent in 2019 (*W* = 705, *p* = 0.057). In addition, browsing intensity significantly decreased in the **∩** treatment between 2009 and 2019 (*W* = 569.5, *p* =  < 0.001). The effect of elevation varied between browsing likelihood and browsing intensity (Supplementary Table 3, Supplementary Fig. 1). Elevation did not affect browsing likelihood in any of the treatments, and this was consistent between 2009 and 2019. Browsing intensity did not vary with elevation in 2009, but significantly decreased with elevation in 2019 for all treatments.

### Birch life-stage prevalence

In total, we registered 704 trees in 2019 (Supplementary Table 7, fewer than the 792 registered in 2009). Between 2009 and 2019, the number of recorded recruits decreased in both the previously no sheep treatment (**∪**) and in the overall ambient sheep density treatment (**Ө**) (Supplementary Table 7). The opposite trends were observed for both saplings and mature trees in the **∪** treatment, where the numbers had increased. However, the same increasing trends for numbers of saplings and mature trees were observed also in the treatment with a previously high sheep density (**∩** treatment).

**Fig. 3:**
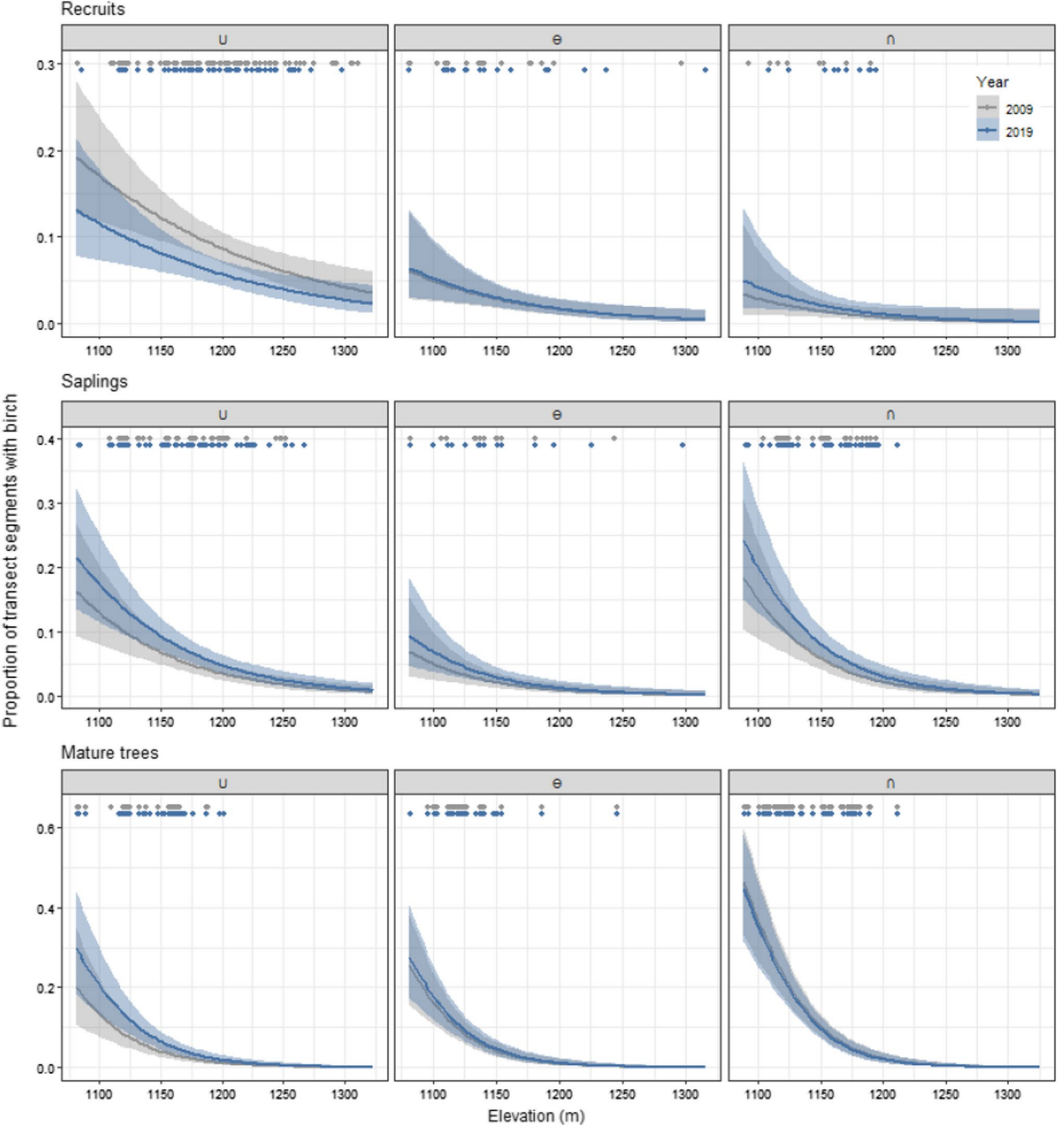
Proportion of transect segments occupied by birch as predicted by the first-ranked logistic regression model in Supplementary Tables 5–6. The predictions are distributed between recruits (height < 175 cm and basal stem diameter ≤ 15 mm), saplings (height < 175 cm and basal stem diameter > 15 mm), and mature trees (height ≥ 175 cm) per year (2009, 2019) for the three different treatments: a decrease followed by a return to ambient sheep density (**∪**), continued ambient density (**Ө**), and an increase followed by a return to ambient sheep density (**∩**). Dotted points at the top of each panel represent transect segments occupied by birch along the elevational gradient for each year

For birch prevalence, three statistical models were identified as being the top-ranked models as they had a ΔAICc < 2 (Supplementary Table 6). The models varied in complexity, with the first and third-ranked models having three-way interactions between year of the data sampling (2009 and 2019), browsing treatment and birch life-stage class. The second-ranked model had no three-way interactions, and also lacked the two-way interactions between data sampling year and treatment, and sampling year and elevation. For the first-ranked model, birch prevalence significantly varied with data sampling year, browsing treatment and elevation (Table 3). For the **∪** treatment, there was a significant decrease in recruit prevalence between 2009 and 2019 (Wilcoxon rank sum test: *W* = 550,088, *p* = 0.01). For all other treatments and birch life-stages, the difference between the years were not significant. However, birch prevalence varied along the elevational gradient between the two years (Fig. 2, 3, Supplementary Table 6). For the **∪** treatment, the probability of finding saplings and mature trees at higher elevations increased between 2009 and 2019 for two of three top-ranked models (Fig. 3, Supplementary Table 6).

**Table 3:** Analysis of deviance for the first-ranked logistic regression model for birch prevalence

Predictors	*Df*	Wald Chisq	*p*
Year	1	5.40	0.02
Treatment	2	75.70	< 0.01
Class	2	2.47	0.29
Elevation	1	332.20	< 0.01
Year × treatment	2	1.49	0.47
Year × class	2	14.89	< 0.01
Treatment × class	4	53.20	< 0.01
Treatment × elevation	2	8.43	0.02
Class × elevation	2	57.52	< 0.01
Year × treatment × class	4	11.68	0.02

Level of significance: *p* ≤ 0.05

### Birch radial growth

**Fig. 4:**
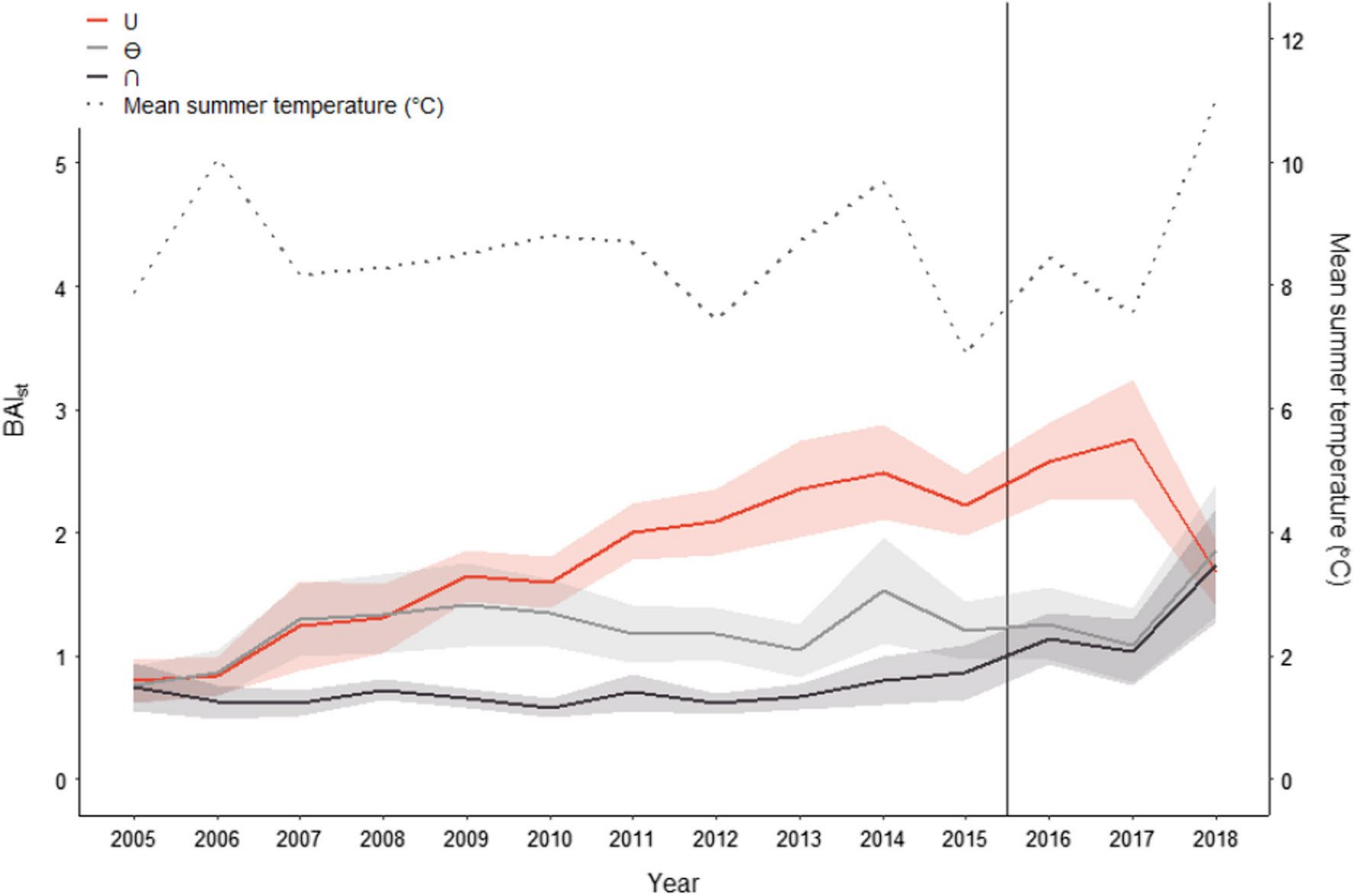
Standardised basal area increment (BAI_st_) per year (2009, 2019) for the three different treatments: a decrease followed by a return to ambient sheep density (**∪**), continued ambient density (**Ө**), and an increase followed by a return to ambient sheep density (**∩**). Solid and shaded area represent the BAI_st_ mean and SE for each treatment, respectively. The dotted line represents the mean summer temperature (°C) per year. The vertical line represents when the enclosures were removed. The erection of the enclosures in 2002 is not included in the figure

The three sheep density treatments showed different trends in standardised basal area increment (BAI_st_) over time (Fig. 4). Between the treatments, BAI_st_ diverged until around the time, where the enclosures were removed and the whole area had an ambient low sheep density. After this, there was a 2-year lag before the growth within the treatments converged.

**Fig. 5:**
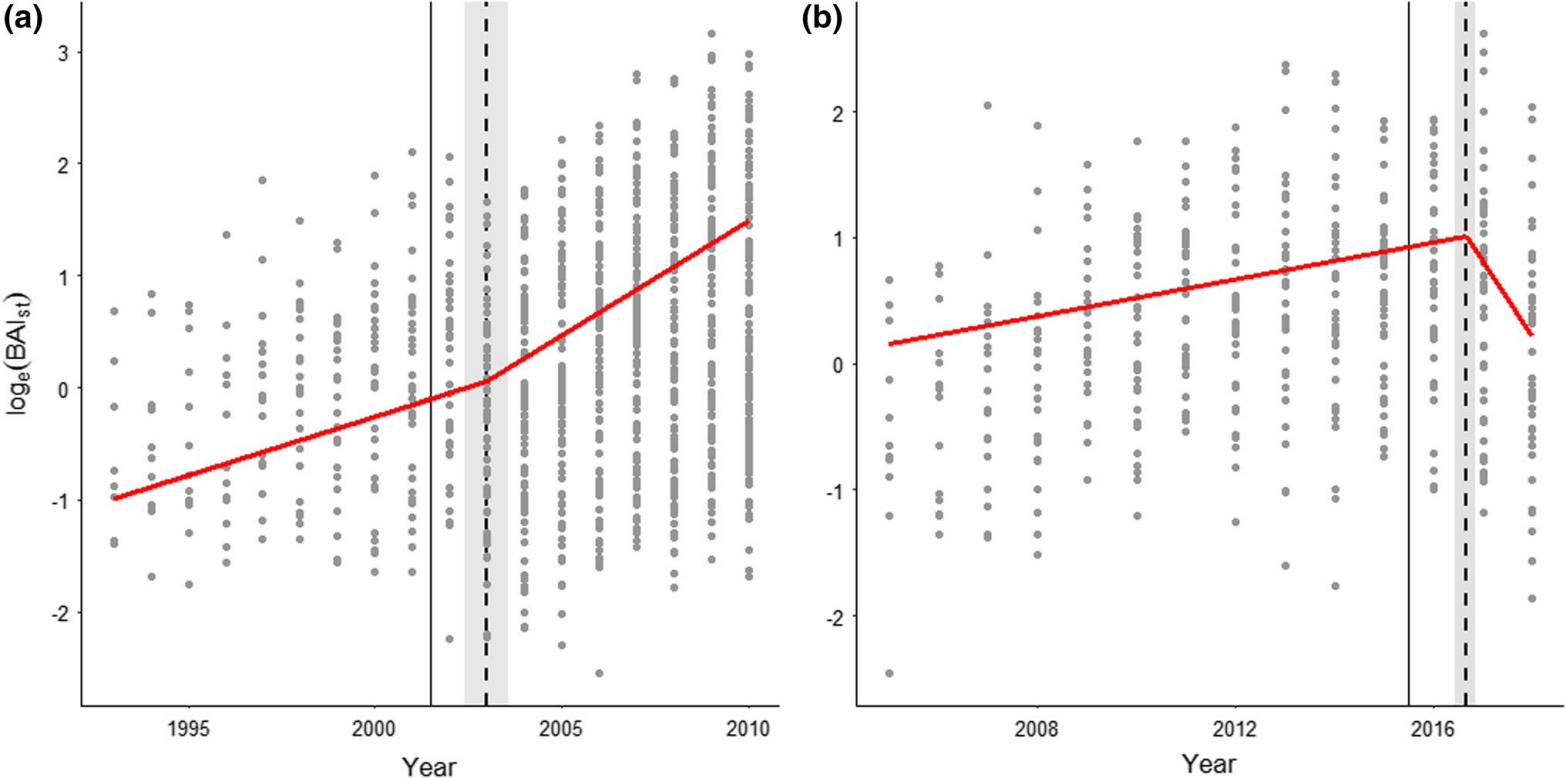
Logarithm of standardised basal area increment (BAI_st_) for the treatment with a decrease followed by a return to ambient sheep density (**∪**) represented by two data series for when the treatment **a** started (2002) and **b** ended (2015) (vertical solid lines). Vertical dashed lines represent the breakpoints in the first-ranked segmented linear mixed-effect model and the shaded area represent the standard error of the breakpoint

For all three treatments, multiple models had ΔAICc < 2, and were thus considered as being top-ranked. For the treatment, where sheep were excluded during the experimental period **(∪)**, all the top-ranked models had significant breakpoints in their temporal series, and the breakpoints did not differ from each other. The first-ranked segmented regression analyses showed that the growth rate in the **∪** treatment, as expressed by the slope parameter of BAI_st_, accelerated around the year 2003 (2003 ± 0.6 year) and decreased around 2016–2017 (2016.65 ± 0.22 year) (Fig. 5). Both before and after the first breakpoint in 2004 (Fig. 5a), BAI_st_ was increasing, but at different rates (0.10 ± 0.02 per year before the removal of sheep and 0.21 ± 0.02 after the removal of sheep). At the second breakpoint in 2017 (Fig. 5b), BAI_st_ changed from neither increasing nor decreasing (0.07 ± 0.07 per year before the return of sheep) to decreasing (− 0.59 ± 0.24 per year after the return of sheep).

For the two treatments with sheep present during the whole experiment (**Ө** and **∩**), there were no significant breakpoints in the temporal series for BAI_st_ around the times the fences were taken down, and these two treatments were thus represented as non-segmented models, where ΔAICc < 2 (Supplementary Tables 11–12). For all the top-ranked models for the first data series (1993–2010), BAI_st_ increased with year in the **Ө** treatment (Supplementary Table 11). Elevation and the interaction between elevation and temperature were also important for BAI_st_, but these variables only occurred in one of four top-ranked models. For the second data series (2005–2018), all the variables were included in at least one of the top-ranked models for the **Ө** treatment. However, of all the variables, only temperature had a significant effect on BAI_st_, and this effect was only present in two of eleven models. For the **∩** treatment for the first data series, BAI_st_ increased with temperature in three of four top-ranked models (Supplementary Table 12). In the second data series for the **∩** treatment, BAI_st_ decreased with elevation in two of three top-ranked models. However, when elevation increased together with temperature, BAI_st_ increased.

## Discussion

Treeline dynamics are affected by complex long-term interacting factors, such as climate and herbivory. In this study, we show how treeline-forming mountain birch respond to experimental changes in sheep density along an elevational gradient in the treeline ecotone. We found that changes in sheep density affect birch trees at different life-stages (recruits, saplings, and mature trees) differently, and that a long-term increase or decrease of sheep densities can lead to alternative successional trajectories towards a forest or alpine ecosystem and thus leave legacy effects.

### Herbivory and birch life-stage prevalence

As predicted (P1), there was no change in browsing likelihood and intensity, or birch prevalence between 2009 and 2019 for the treatment that had a continuous ambient low sheep density throughout the study period (**Ө** treatment). This treatment can thus be considered a control. When sheep were excluded and then reintroduced to an ambient low density (**∪** treatment), the browsing likelihood and intensity increased at low elevations, and recruits became less prevalent. This was according to our prediction (P2), and shows that there were no legacy effects on the number of birch recruits after 14 years without sheep. Saplings on the other hand, had a higher prevalence in the **∪** treatment in 2019. This shows that it is mainly recently recruited trees (basal stem diameter ≤ 15 mm) that are negatively affected by browsing, even though both recruits and saplings are within the heights of being browsed by sheep (height < 175 cm). Thus, birch saplings in the treeline ecotone seem to have higher resistance to herbivory than recruits, possibly due to higher resource storage in the roots (Boege and Marquis 2005). In addition, increased levels of condensed tannins in birch foliage with age may prevent browsing on birch (Wam et al. 2017).

In contrast to our predictions (P3) for the treatment, where sheep density was increased and then decreased (**∩** treatment), there were no significant changes in recruitment or sapling prevalence for this treatment between the 2 years, suggesting that 14 years with high sheep densities are still observable 4 years after a reduction in sheep density. These results are in line with Speed et al. (2010), who showed that even low sheep densities limited birch recruitment. Eight years after the experiment started in 2002, the probability of finding recruits was much higher in the absence of sheep than in enclosures with sheep (Speed et al. 2010). In our study, browsing likelihood did not vary between the two treatments with sheep present during the whole experiment (**Ө** and **∩**), but the intensity of the browsing decreased in the **∩** treatment between 2009 and 2019. In addition, the browsing intensity in the **∩** treatment became more similar to the ambient sheep treatment (**Ө**) after the experiment ended. This implies that it is the presence of sheep, even with low densities, that limits recruitment, and that a decrease in sheep density from high to low level as in the **∩** treatment, does not necessarily increase recruit prevalence. However, recruit prevalence in the **∩** treatment was more similar to the **Ө** treatment in 2019 than in 2009, even though the number of recruits had not changed between the years. Recruitment is expected to be higher for a limited period after grazing cessation due to a combination of lower browsing pressure (i.e., window of opportunity; cf. Didion et al. 2009) and lower competition from surrounding vegetation that was suppressed by the prior high sheep density and has not regenerated (Austrheim et al. 2014).

The number of mature trees had increased between 2009 and 2019 in the **∪** treatment, and two of three of the top-ranked models in this study showed an increased prevalence of mature trees between the 2 years (consistent with P4). An increased density of mature trees indicates that the **∪** treatment may be entering an alternative successional trajectory compared to the **Ө** treatment with a continuous ambient sheep density. This is likely because mature trees do not disappear with increased browsing pressure as they have reached a height, where parts of the individuals escape sheep browsing (≥ 175 cm) (Speed et al. 2011a). This can also be seen in the **∩** treatment, where there was no change in the prevalence of mature trees in both 2009 and 2019. The **∪** treatment thus shows tendencies to legacy effects after 14 years without sheep being present. An alternative successional trajectory triggered by a lack of browsing by sheep could lead the **∪** treatment into an alternative stable state if no other disturbances affect mature trees. Within the treeline ecotone, an alternative to the open, alpine landscapes could be a forest, particularly in a warming climate. The establishment of pioneer trees above the treeline can facilitate further tree establishment (Germino et al. 2002). This will give a positive feedback from the cohort of trees establishing during the window of opportunity opened by sheep absence, thus leading to further afforestation. Many European mountains have in the last decades experienced lowered densities of livestock due to less pasture activities (MacDonald et al. 2000; Ross et al. 2016). As we have found in this study, browsing on recruits can regulate the number of mature trees, and may explain why many European mountains with previously high livestock densities are experiencing tree densification and upward treeline shifts (Hofgaard 1997; Gehrig‐Fasel et al. 2007; Ameztegui et al. 2016; Piccinelli et al. 2020).

Our results thus suggest that birch life-stages are differently affected by browsing, and the treeline is then also affected accordingly. However, potential forest expanse into the treeline ecotone will depend on the treeline forming species and type of herbivore. Fast-growing species such as birch will likely recover faster from browsing than slow-growing coniferous species, such as spruce and pine. Trees with high palatability has been found to have higher growth rates than non-palatable species (Bee et al. 2007), thus being able to compensate for browsing. Different herbivores may also affect the trees differently. Smaller mammals such as hares may browse on shorter conifers and thus inhibit succession and treeline expansion (Olnes and Kielland 2016; Olnes et al. 2017). Larger mammals can both buffer (Cairns and Moen 2004) and facilitate (Tømmervik et al. 2004) treeline expansions due to impacts on the trees and competing vegetation, respectively. Local outbreaks of insect defoliators can reset decades of treeline expanse, most likely due to climate warming (Jepsen et al. 2008). Thus, potential treeline advance is context dependent as treeline forming species and herbivore species vary between localities.

### Herbivory and birch radial growth

Herbivory is often found to override the impact of temperature in determining tree radial growth (Speed et al. 2011b; Fisichelli et al. 2012; Herrero et al. 2016). In our study, we found radial growth represented as standardised basal area increment (BAI_st_), to be affected likewise. As predicted (P5), radial growth increased in the **∪** treatment 2 years after sheep were removed and enclosures erected, and decreased 2 years after enclosure removal and sheep density returned to ambient low density. Thus, tree radial growth does not immediately respond to a change in herbivore densities, and legacy effects were present for 2 years creating a lag effect. A lag effect in this context can be explained by trees having enough reserves to withstand 1 or 2 years of browsing, but after 2 years the trees allocate less resources to radial growth. We also observed an increase in radial growth for the **∩** treatment, thus confirming our prediction (P5). However, this increase did not show as a threshold response as expected but generally increased over the years. This could imply that the lowered sheep density after the enclosures were removed had a more gradual positive effect on radial growth than the abrupt negative effect of the increased sheep density after enclosure removal in the **∪** treatment. A similar pattern was seen in Speed et al. (2011b), where there was a gradual increase in radial growth for the no sheep treatment after the enclosures were erected.

### Herbivory, temperature, and treeline advance

In general, temperature decreases with increasing elevation, and temperature is often found to be one of the main drivers of treeline dynamics (Körner and Paulsen 2004; Smith et al. 2009; Mienna et al. 2020). Birch prevalence decreased with increasing elevation for all three life-stages. As the interaction between elevation and year was not found significant for the prevalence of any birch life-stage, there was no change in where birch trees were found along the elevational gradient between 2009 and 2019. Thus, the change in recruit and sapling prevalence for the **∪** treatment was independent of elevation. This is concordant with our results for radial growth in the same treatment. The fact that elevation did not have a significant effect on either prevalence or radial growth of recruits or saplings in the **∪** treatment could suggest that sheep browsing overruled the potential temperature effect of elevation after the enclosures were removed, as shown during 2002–2010 (Speed et al. 2011b). Browsing has previously been found to remove positive temperature effects on sapling radial growth (Fisichelli et al. 2012; Vuorinen et al. 2020), supporting our results. Thus, browsing seem to affect growth of birch trees more than temperature, at least for small trees, such as recruits and saplings. Air temperature during early life-stages of treeline trees may be less important due to the more important role of interactions with other plants (Tingstad et al. 2015). However, when the tree reaches a certain height, the surrounding temperature will most likely be more important for determining tree growth and establishment as mature trees (Körner 2012).

In the 30 year period between 1989 and 2018, the mean annual temperature increased with 0.93 °C at the experimental site. Warmer temperatures and lower browsing pressure by herbivores should provide better growing conditions, leading the trees to grow taller until they escape potential browsing, as shown in this study for the **∪** treatment. As temperature is thought to be a major driver of treeline dynamics, an increase in temperature should lead to higher treeline elevation. In this study, treeline elevation (highest elevation for trees > 2 m) had increased by 10 m between 2009 and 2019 for the treatment that had 14 years with no sheep and then an increase in sheep density (**∪** treatment). For the two other treatments, where sheep had been present throughout the whole study period (**Ө, ∩**), there had been no change in treeline elevation. An annual elevational shift of 1 m per year is concordant with elevational shifts of forest lines (Bryn and Potthoff 2018) and plant communities (Klanderud and Birks 2003; Speed et al. 2012) in Norway. However, as found in our study, herbivores may inhibit treeline advance by lowering tree establishment rates (Speed et al. 2010; Bello-Rodriguez et al. 2019) and growth (Bognounou et al. 2018; Olnes et al. 2018) and thus overrule climate warming effects. Legacies of herbivory could explain the lack of treeline advance many places in Norway, where previous land use still affects current treeline elevations (Bryn and Potthoff 2018).

### Conclusions

We have shown that after four seasons of ambient low densities of sheep, legacy effects and alternative successional trajectories exist following 14 years of experimentally increased or reduced sheep densities. Our results indicate that 4 years of increased sheep densities in areas, where sheep were previously excluded (**∪** treatment) have negative effects for the growth and survival of birch recruits. However, saplings and mature trees are less affected, and will most likely be able to escape future herbivore induced mortality by sheep. The prevalence of mature trees had indeed increased during the period without sheep, and thus possibly entered an alternative successional trajectory compared to areas that had continuous ambient sheep density (**Ө** treatment). Prevalence and radial growth of recruits and saplings are more affected by browsing than by temperature. In conclusion, birch tree persistence after experimentally increased or decreased sheep browsing are depending on tree life-stage. Temporal changes in herbivore densities within the treeline ecotone can, therefore, direct the ecosystem into alternative successional trajectories.

## Supplementary Information

Below is the link to the electronic supplementary material.

Supplementary file1 (DOCX 2169 kb)

## Data Availability

The data that support the findings of this study is submitted to a public repository. Questions concerning the data can be addressed to the corresponding author.

## Code availability

The R script that can be used to reproduce the findings of this study is submitted to a public repository. Questions concerning the R script can be addressed to the corresponding author.
