# Supplementary material for: Legacy effects of herbivory on treeline dynamics along an elevational gradient
Source: Oecologia. 2022 Feb 11;198(3):801–14. doi: 10.1007/s00442-022-05125-8 (PMC8956534; doi:10.1007/s00442-022-05125-8)
Supplement: Supplementary file 1 — Supplementary file1 (DOCX 2169 kb) [file 442_2022_5125_MOESM1_ESM.docx]

Supplementary tables

Supplementary Table 1. All browsing likelihood models ranked from lowest conditional Akaike Information Criterion (AICc) to highest. The top-ranked model (ΔAICc < 2) is marked in bold. The model terms are: 1, Elevation; 2, Treatment; 3, Year; 4, Elevation × Treatment; 5, Elevation × Year; 6, Treatment × Year; 7, Elevation × Treatment × Year.

| *Rank* | *Models* | *Df* | *logLik* | *AICc* | *ΔAICc* | *AICc weights* |
| --- | --- | --- | --- | --- | --- | --- |
| **1** | **1/2/3/4/5/6/7** | **12** | **-441.352** | **907.029** | **0** | **0.749** |
| 2 | 1/2/3/4/5/6 | 10 | -445.099 | 910.427 | 3.398 | 0.137 |
| 3 | 1/2/3/5/6 | 8 | -447.419 | 910.987 | 3.958 | 0.104 |
| 4 | 2/3/6 | 6 | -452.448 | 916.983 | 9.954 | 0.005 |
| 5 | 1/2/3/4/6 | 9 | -449.894 | 917.975 | 10.946 | 0.003 |
| 6 | 1/2/3/6 | 7 | -452.432 | 918.981 | 11.952 | 0.002 |
| 7 | 1/2/3/4/5 | 8 | -459.089 | 934.328 | 27.299 | 0 |
| 8 | 1/2/4 | 6 | -462.655 | 937.397 | 30.368 | 0 |
| 9 | 1/2/3/4 | 7 | -462 | 938.116 | 31.086 | 0 |
| 10 | 1/2/3/5 | 6 | -463.256 | 938.599 | 31.57 | 0 |
| 11 | 2/3 | 4 | -466.943 | 941.928 | 34.899 | 0 |
| 12 | 2 | 3 | -467.968 | 941.961 | 34.932 | 0 |
| 13 | 1/2/3 | 5 | -466.748 | 943.558 | 36.529 | 0 |
| 14 | 1/2 | 4 | -467.831 | 943.704 | 36.675 | 0 |
| 15 | 1/3/5 | 4 | -499.638 | 1007.318 | 100.289 | 0 |
| 16 | 1 | 2 | -503.303 | 1010.618 | 103.588 | 0 |
| 17 | 1/3 | 3 | -502.685 | 1011.396 | 104.366 | 0 |
| 18 | (Null) | 1 | -505.716 | 1013.436 | 106.407 | 0 |
| 19 | 3 | 2 | -505.309 | 1014.63 | 107.601 | 0 |

Supplementary Table 2. All browsing intensity candidate models ranked from lowest conditional Akaike Information Criterion (AICc) to highest. The top-ranked model (ΔAICc < 2) is marked in bold. The model terms are: 1, Elevation; 2, Treatment; 3, Year; 4, Elevation × Treatment; 5, Elevation × Year; 6, Treatment × Year; 7, Elevation × Treatment × Year.

| *Rank* | *Models* | *Df* | *logLik* | *AICc* | *ΔAICc* | *AICc weights* |
| --- | --- | --- | --- | --- | --- | --- |
| **1** | **1/2/3/5/6** | **9** | **75.811** | **-133.434** | **0** | **0.632** |
| 2 | 1/2/3/4/5/6/7 | 13 | 78.678 | -130.976 | 2.459 | 0.185 |
| 3 | 1/2/3/4/5/6 | 11 | 76.035 | -129.794 | 3.64 | 0.102 |
| 4 | 2/3/6 | 7 | 71.332 | -128.547 | 4.887 | 0.055 |
| 5 | 1/2/3/6 | 8 | 71.404 | -126.658 | 6.776 | 0.021 |
| 6 | 1/2/3/4/6 | 10 | 71.734 | -123.238 | 10.197 | 0.004 |
| 7 | 1/2/3/4/5 | 9 | 39.378 | -60.569 | 72.866 | 0 |
| 8 | 1/2/4 | 7 | 36.152 | -58.187 | 75.247 | 0 |
| 9 | 1/2/3/4 | 8 | 37.083 | -58.017 | 75.418 | 0 |
| 10 | 1/2/3/5 | 7 | 34.139 | -54.162 | 79.272 | 0 |
| 11 | 2 | 4 | 30.327 | -52.613 | 80.821 | 0 |
| 12 | 2/3 | 5 | 30.994 | -51.926 | 81.509 | 0 |
| 13 | 1/2 | 5 | 30.966 | -51.869 | 81.565 | 0 |
| 14 | 1/2/3 | 6 | 31.532 | -50.977 | 82.458 | 0 |
| 15 | 1/3/5 | 5 | -13.439 | 36.941 | 170.375 | 0 |
| 16 | 1 | 3 | -15.815 | 37.655 | 171.089 | 0 |
| 17 | 1/3 | 4 | -14.978 | 37.997 | 171.431 | 0 |
| 18 | 3 | 3 | -17.674 | 41.372 | 174.807 | 0 |
| 19 | (Null) | 2 | -18.814 | 41.641 | 175.075 | 0 |

Supplementary Table 3. Parameter estimates of the top-ranked (ΔAICc < 2) logistic regression model for browsing likelihood and linear regression model for browsing intensity of birch trees < 175 cm in height and < 30 mm in basal stem diameter. Elevation starts at 1081 m. Level of significance: *p* ≤ 0.05.

|  | *Browsing likelihood* | | |  | *Browsing intensity* | | |
| --- | --- | --- | --- | --- | --- | --- | --- |
| *Predictors* | *Coefficient* | *std. Error* | *p* |  | *Coefficient* | *std. Error* | *p* |
| (Intercept) | 0.291 | 0.385 | 0.450 |  | 0.247 | 0.033 | **<0.001** |
| Year (2019) | -0.812 | 0.588 | 0.164 |  | -0.001 | 0.048 | 0.843 |
| **U** | -2.565 | 0.492 | **<0.001** |  | -0.224 | 0.032 | **<0.001** |
| **ꓵ** | 1.400 | 1.278 | 0.273 |  | 0.318 | 0.052 | **<0.001** |
| Elevation | 0.002 | 0.005 | 0.676 |  | 0.000 | 0.000 | 0.127 |
| Year (2019) × **U** | 2.324 | 0.740 | **0.002** |  | 0.160 | 0.048 | **0.001** |
| Year (2019) × **ꓵ** | 6.667 | 6.381 | 0.296 |  | -0.419 | 0.077 | **<0.01** |
| Year (2019) × Elevation | -0.001 | 0.008 | 0.879 |  | -0.001 | 0.000 | **0.003** |
| **U** × Elevation | 0.004 | 0.006 | 0.464 |  |  |  |  |
| **ꓵ** × Elevation | -0.007 | 0.018 | 0.680 |  |  |  |  |
| Year (2019) × **U** × Elevation | -0.015 | 0.010 | 0.109 |  |  |  |  |
| Year (2019) × **ꓵ** × Elevation | -0.195 | 0.162 | 0.231 |  |  |  |  |
|  |  |  |  |  |  |  |  |
| Pseudo *R^2^*: 0.13 |  |  |  |  | Adj. *R^2^*: 0.17 | | |
|  |  |  |  |  | F_7,963_: 29.6, *p* <0.01 | | |

Supplementary Table 4. Estimated detection probabilities of recruits and saplings per treatment (**ꓴ, Ө, ꓵ**) per year (2009, 2019). We used the probabilities as model offsets when evaluating change in birch prevalence. We assumed mature trees to have a detection probability of 1.

|  | **ꓴ** | |  | **Ө** | |  | **ꓵ** | |
| --- | --- | --- | --- | --- | --- | --- | --- | --- |
|  | 2009 | 2019 |  | 2009 | 2019 |  | 2009 | 2019 |
| Recruit | 0.999 | 0.999 |  | 1 | 0.522 |  | 0.711 | 0.595 |
| Sapling | 1 | 0.556 |  | 0.999 | 0.767 |  | 0.959 | 0.868 |

Supplementary Table 5. A 95% confidence set of the best-ranked birch prevalence candidate models. The top-ranked models (ΔAICc < 2) are marked in bold. The model terms are: 1, Class; 2, Elevation; 3, Treatment; 4, Year; 5, Class × Elevation; 6, Class × Treatment; 7, Class × Year: 8, Elevation × Treatment; 9, Elevation × Year; 10, Treatment × Year; 11, Class × Elevation × Treatment; 12, Class × Elevation × Year; 13, Elevation × Treatment × Year; 14, Class × Elevation × Treatment × Year.

| *Rank* | *Models* | *Df* | *logLik* | *AICc* | *ΔAICc* | *AICc weights* |
| --- | --- | --- | --- | --- | --- | --- |
| **1** | **1/2/3/4/5/6/7/8/10/13** | **23** | **-2308.755** | **4663.576** | **0** | **0.297** |
| **2** | **1/2/3/4/5/6/7/8** | **17** | **-2315.384** | **4664.805** | **1.228** | **0.161** |
| **3** | **1/2/3/4/5/6/7/8/9/10/13** | **24** | **-2308.69** | **4665.451** | **1.875** | **0.116** |
| 4 | 1/2/3/4/5/6/7/8/9 | 18 | -2315.176 | 4666.393 | 2.817 | 0.073 |
| 5 | 1/2/3/4/5/6/7/8/10/11/13 | 27 | -2306.296 | 4666.681 | 3.105 | 0.063 |
| 6 | 1/2/3/4/5/6/7/8/10 | 19 | -2314.663 | 4667.371 | 3.795 | 0.045 |
| 7 | 1/2/3/4/5/6/7/8/11 | 21 | -2312.933 | 4667.922 | 4.345 | 0.034 |
| 8 | 1/2/3/4/5/6/7/8/9/10/13/14 | 26 | -2307.969 | 4668.022 | 4.445 | 0.032 |
| 9 | 1/2/3/4/5/6/7/10/13 | 21 | -2313.049 | 4668.154 | 4.577 | 0.03 |
| 10 | 1/2/3/4/5/6/7/8/9/10/11/13 | 28 | -2306.265 | 4668.626 | 5.05 | 0.024 |
| 11 | 1/2/3/4/5/6/7/8/9/12 | 20 | -2314.371 | 4668.793 | 5.216 | 0.022 |
| 12 | 1/2/3/4/5/6/7/8/9/10 | 20 | -2314.516 | 4669.082 | 5.505 | 0.019 |
| 13 | 1/2/3/4/5/6/7/8/9/10/12/13 | 26 | -2308.509 | 4669.101 | 5.524 | 0.019 |
| 14 | 1/2/3/4/5/6/7/8/9/11 | 22 | -2312.643 | 4669.345 | 5.769 | 0.017 |
| 15 | 1/2/3/4/5/6/7 | 15 | -2319.77 | 4669.569 | 5.993 | 0.015 |
| 16 | 1/2/3/4/5/6/7/8/9/10/14 | 22 | -2312.799 | 4669.659 | 6.082 | 0.014 |
| 17 | 1/2/3/4/5/6/7/9/10/13 | 22 | -2312.956 | 4669.973 | 6.397 | 0.012 |
| 18 | 1/2/3/4/5/6/7/8/10/11 | 23 | -2312.222 | 4670.509 | 6.932 | 0.009 |

Supplementary Table 6. Parameter estimates of the top-ranked (ΔAICc < 2) linear regression models for birch prevalence. Elevation starts at 1081 m. Level of significance: p ≤ 0.05.

| *Predictors* | *Model 1* | *Model 2* | *Model 3* |
| --- | --- | --- | --- |
| (Intercept) | -3.729 ± 0.301 *** | -3.425 ± 0.254 *** | -3.754 ± 0.31 *** |
| Year (2019) | 0.534 ± 0.334 | -0.165 ± 0.147 | 0.588 ± 0.365 |
| **U** | 1.292 ± 0.335 *** | 0.86 ± 0.279 ** | 1.287 ± 0.336 *** |
| **ꓵ** | -0.242 ± 0.486 | -0.185 ± 0.362 | -0.239 ± 0.487 |
| Sapling | 0.144 ± 0.4 | -0.191 ± 0.309 | 0.144 ± 0.401 |
| Mature tree | 1.679 ± 0.349 *** | 1.363 ± 0.285 *** | 1.687 ± 0.35 *** |
| Elevation | -0.012 ± 0.002 *** | -0.012 ± 0.002 *** | -0.011 ± 0.002 *** |
| Year (2019) × **U** | -0.988 ± 0.378 ** |  | -0.971 ± 0.381 * |
| Year (2019) × **ꓵ** | -0.006 ± 0.569 |  | -0.009 ± 0.569 |
| **U** × Elevation | 0.004 ± 0.002 | 0.004 ± 0.002 | 0.004 ± 0.002 |
| **ꓵ** × Elevation | -0.003 ± 0.003 | -0.003 ± 0.003 | -0.003 ± 0.003 |
| Year (2019) × Sapling | 0.036 ± 0.499 | 0.791 ± 0.209 *** | 0.025 ± 0.5 |
| Year (2019) × Mature tree | -0.423 ± 0.429 | 0.299 ± 0.205 | -0.446 ± 0.433 |
| Year (2019) × Elevation |  |  | -0.001 ± 0.002 |
| **U** × Sapling | -0.342 ± 0.425 | 0.209 ± 0.292 | -0.345 ± 0.425 |
| **ꓵ** × Sapling | 1.526 ± 0.547 ** | 1.393 ± 0.359 *** | 1.523 ± 0.547 ** |
| **U** × Mature tree | -1.615 ± 0.407 *** | -0.959 ± 0.287 *** | -1.614 ± 0.407 *** |
| **ꓵ** × Mature tree | 1.372 ± 0.5 ** | 1.227 ± 0.339 *** | 1.366 ± 0.5 ** |
| Sapling × Elevation | -0.006 ± 0.002 ** | -0.006 ± 0.002 ** | -0.006 ± 0.002 ** |
| Mature tree × Elevation | -0.018 ± 0.002 *** | -0.018 ± 0.002 *** | -0.018 ± 0.002 *** |
| Year (2019) × **U** × Sapling | 1.206 ± 0.571 * |  | 1.206 ± 0.571 * |
| Year (2019) × **ꓵ** × Sapling | -0.125 ± 0.721 |  | -0.119 ± 0.721 |
| Year (2019) × **U** × Mature tree | 1.39 ± 0.544 * |  | 1.384 ± 0.545 * |
| Year (2019) × **ꓵ** × Mature tree | -0.171 ± 0.663 |  | -0.159 ± 0.664 |
|  |  |  |  |
| Pseudo *R^2^* | 0.142 | 0.142 | 0.144 |

Supplementary Table 7. Number of trees classed as recruits (height < 175 cm and basal stem diameter ≤ 15 mm), saplings (height < 175 cm and basal stem diameter > 15 mm), and mature trees (height ≥ 175 cm) per treatment (**ꓴ,** **Ө** and **ꓵ**) recorded along the same 15 transects in 2009 and 2019.

|  | 2009 / 2019 | | |
| --- | --- | --- | --- |
|  | **ꓴ** | **Ө** | **ꓵ** |
| Recruits | 470 / 271 | 47 / 39 | 15 / 15 |
| Saplings | 43 / 94 | 20 / 20 | 48 / 56 |
| Mature trees | 41 / 65 | 52 / 49 | 92 / 95 |

Supplementary Table 8. All candidate models for birch tree ring increment in the **ꓴ** treatment in the first (1993-2010) and second (2005-2018) data series. The top-ranked models (ΔAICc < 2) are marked in bold. The models are ranked from lowest conditional Akaike Information Criterion (AICc) to highest. The model terms are: 1, Elevation; 2, Temperature; 3, Year; 4, Elevation × Temperature; 5, Elevation × Year; 6, Temperature × Year; 7, Elevation × Temperature × Year.

| 1993 – 2010 | |  |  |  |  |  |  | 2005 – 2018 | |  |  |  |  |  |
| --- | --- | --- | --- | --- | --- | --- | --- | --- | --- | --- | --- | --- | --- | --- |
| *Rank* | *Models* | *Df* | *logLik* | *AICc* | *ΔAICc* | *AICc weights* |  | *Rank* | *Models* | *Df* | *logLik* | *AICc* | *ΔAICc* | *AICc weights* |
| **1** | **1/2/3/4/5** | **9** | **-687.833** | **1393.847** | **0** | **0.298** |  | **1** | **1/2/3/6** | **8** | **-367.398** | **751.135** | **0** | **0.405** |
| **2** | **1/2/3/5** | **8** | **-689.061** | **1394.267** | **0.42** | **0.242** |  | **2** | **1/2/3/5/6** | **9** | **-367.229** | **752.883** | **1.748** | **0.169** |
| **3** | **1/3/5** | **7** | **-690.781** | **1395.674** | **1.827** | **0.12** |  | 3 | 2/3/6 | 7 | -369.46 | 753.182 | 2.047 | 0.145 |
| **4** | **1/2/3/4/5/6/7** | **11** | **-686.75** | **1395.766** | **1.919** | **0.114** |  | 4 | 1/2/3/4/6 | 9 | -367.39 | 753.205 | 2.071 | 0.144 |
| **5** | **1/2/3/4/5/6** | **10** | **-687.798** | **1395.818** | **1.97** | **0.111** |  | 5 | 1/2/3/4/5/6 | 10 | -367.229 | 754.979 | 3.844 | 0.059 |
| 6 | 1/2/3/5/6 | 9 | -688.937 | 1396.056 | 2.209 | 0.099 |  | 6 | 1/2/3/4/5/6/7 | 11 | -366.952 | 756.53 | 5.396 | 0.027 |
| 7 | 1/2/3 | 7 | -693.93 | 1401.972 | 8.125 | 0.005 |  | 7 | 1/2/3 | 7 | -371.316 | 756.895 | 5.76 | 0.023 |
| 8 | 1/2/3/4 | 8 | -693.095 | 1402.334 | 8.487 | 0.004 |  | 8 | 1/2/3/5 | 8 | -371.184 | 758.707 | 7.572 | 0.009 |
| 9 | 1/3 | 6 | -695.739 | 1403.563 | 9.715 | 0.002 |  | 9 | 1/2/3/4 | 8 | -371.314 | 758.966 | 7.831 | 0.008 |
| 10 | 1/2/3/6 | 8 | -693.856 | 1403.858 | 10.01 | 0.002 |  | 10 | 2/3 | 6 | -373.409 | 759.015 | 7.88 | 0.008 |
| 11 | 1/2/3/4/6 | 9 | -693.078 | 1404.336 | 10.489 | 0.002 |  | 11 | 1/2/3/4/5 | 9 | -371.184 | 760.792 | 9.657 | 0.003 |
| 12 | 2/3 | 6 | -699.916 | 1411.917 | 18.07 | 0 |  | 12 | 1/3 | 6 | -377.663 | 767.523 | 16.389 | 0 |
| 13 | 3 | 5 | -701.728 | 1413.516 | 19.669 | 0 |  | 13 | 1/3/5 | 7 | -377.563 | 769.389 | 18.254 | 0 |
| 14 | 2/3/6 | 7 | -699.84 | 1413.793 | 19.945 | 0 |  | 14 | 3 | 5 | -379.74 | 769.62 | 18.485 | 0 |
| 15 | 1/2/4 | 7 | -748.84 | 1511.792 | 117.945 | 0 |  | 15 | 1/2 | 6 | -381.312 | 774.821 | 23.686 | 0 |
| 16 | 1/2 | 6 | -750.041 | 1512.167 | 118.32 | 0 |  | 16 | 1/2/4 | 7 | -381.294 | 776.852 | 25.717 | 0 |
| 17 | 1 | 5 | -753.804 | 1517.667 | 123.82 | 0 |  | 17 | 2 | 5 | -383.442 | 777.025 | 25.89 | 0 |
| 18 | 2 | 5 | -755.183 | 1520.425 | 126.578 | 0 |  | 18 | 1 | 5 | -384.481 | 779.103 | 27.968 | 0 |
| 19 | (Null) | 4 | -758.899 | 1525.837 | 131.99 | 0 |  | 19 | (Null) | 4 | -386.587 | 781.267 | 30.133 | 0 |

Supplementary Table 9. All candidate models for birch standardised basal area increment (BAI_st_) in the **Ө** treatment in the first (1993-2010) and second (2005-2018) data series. The top-ranked models (ΔAICc < 2) are marked in bold. The models are ranked from lowest conditional Akaike Information Criterion (AICc) to highest. The model terms are: 1, Elevation; 2, Temperature; 3, Year; 4, Elevation × Temperature; 5, Elevation × Year; 6, Temperature × Year; 7, Elevation × Temperature × Year.

| 1993 – 2010 | |  |  |  |  |  |  | 2005 – 2018 | |  |  |  |  |  |
| --- | --- | --- | --- | --- | --- | --- | --- | --- | --- | --- | --- | --- | --- | --- |
| *Rank* | *Models* | *Df* | *logLik* | *AICc* | *ΔAICc* | *AICc weights* |  | *Rank* | *Models* | *Df* | *logLik* | *AICc* | *ΔAICc* | *AICc weights* |
| **1** | **3** | **5** | **-376.881** | **763.874** | **0** | **0.252** |  | **1** | **2/3/6** | **7** | **-235.622** | **485.717** | **0** | **0.113** |
| **2** | **1/2/3/4** | **8** | **-374.508** | **765.283** | **1.409** | **0.125** |  | **2** | **1/2/3/4/6** | **9** | **-233.618** | **486.002** | **0.285** | **0.098** |
| **3** | **1/3** | **6** | **-376.579** | **765.313** | **1.439** | **0.123** |  | **3** | **1/2/4** | **7** | **-235.785** | **486.043** | **0.326** | **0.096** |
| **4** | **2/3** | **6** | **-376.833** | **765.821** | **1.947** | **0.095** |  | **4** | **2** | **5** | **-237.931** | **486.114** | **0.397** | **0.093** |
| 5 | 1/2/3/4/5 | 9 | -373.828 | 765.993 | 2.119 | 0.087 |  | **5** | **2/3** | **6** | **-237.124** | **486.6** | **0.883** | **0.073** |
| 6 | 1/3/5 | 7 | -375.971 | 766.15 | 2.276 | 0.081 |  | **6** | **1/2/3/4** | **8** | **-235.009** | **486.628** | **0.911** | **0.072** |
| 7 | 1/2/3 | 7 | -376.531 | 767.27 | 3.396 | 0.046 |  | **7** | **3** | **5** | **-238.236** | **486.722** | **1.005** | **0.068** |
| 8 | 1/2/3/4/6 | 9 | -374.473 | 767.282 | 3.408 | 0.046 |  | **8** | **(Null)** | **4** | **-239.461** | **487.089** | **1.372** | **0.057** |
| 9 | 2/3/6 | 7 | -376.833 | 767.873 | 3.999 | 0.034 |  | **9** | **1/2/3/6** | **8** | **-235.379** | **487.369** | **1.652** | **0.049** |
| 10 | 1/2/3/4/5/6 | 10 | -373.806 | 768.023 | 4.149 | 0.032 |  | **10** | **1/2/3/4/5/6/7** | **11** | **-232.119** | **487.371** | **1.654** | **0.049** |
| 11 | 1/2/3/5 | 8 | -375.912 | 768.092 | 4.218 | 0.031 |  | **11** | **1/2** | **6** | **-237.656** | **487.666** | **1.949** | **0.043** |
| 12 | 1/2/3/4/5/6/7 | 11 | -373.176 | 768.846 | 4.972 | 0.021 |  | 12 | 1/2/3/4/5/6 | 10 | -233.608 | 488.157 | 2.44 | 0.033 |
| 13 | 1/2/3/6 | 8 | -376.531 | 769.33 | 5.456 | 0.016 |  | 13 | 1/2/3 | 7 | -236.882 | 488.237 | 2.52 | 0.032 |
| 14 | 1/2/3/5/6 | 9 | -375.911 | 770.157 | 6.284 | 0.011 |  | 14 | 1/3 | 6 | -237.999 | 488.35 | 2.633 | 0.03 |
| 15 | (Null) | 4 | -386.191 | 780.457 | 16.583 | 0 |  | 15 | 1 | 5 | -239.183 | 488.618 | 2.901 | 0.026 |
| 16 | 1 | 5 | -385.931 | 781.972 | 18.099 | 0 |  | 16 | 1/2/3/4/5 | 9 | -235 | 488.767 | 3.05 | 0.025 |
| 17 | 2 | 5 | -386.184 | 782.479 | 18.605 | 0 |  | 17 | 1/2/3/5/6 | 9 | -235.264 | 489.293 | 3.576 | 0.019 |
| 18 | 1/2/4 | 7 | -384.159 | 782.527 | 18.653 | 0 |  | 18 | 1/2/3/5 | 8 | -236.767 | 490.144 | 4.427 | 0.012 |
| 19 | 1/2 | 6 | -385.923 | 784.001 | 20.127 | 0 |  | 19 | 1/3/5 | 7 | -237.874 | 490.221 | 4.504 | 0.012 |

Supplementary Table 10. All candidate models for birch standardised basal area increment (BAI_st_) in the **ꓵ** treatment in the first (1993-2010) and second (2005-2018) data series. The top-ranked models (ΔAICc < 2) are marked in bold. The models are ranked from lowest conditional Akaike Information Criterion (AICc) to highest. The model terms are: 1, Elevation; 2, Temperature; 3, Year; 4, Elevation × Temperature; 5, Elevation × Year; 6, Temperature × Year; 7, Elevation × Temperature × Year.

| 1993 – 2010 | |  |  |  |  |  |  | 2005 – 2018 | |  |  |  |  |  |
| --- | --- | --- | --- | --- | --- | --- | --- | --- | --- | --- | --- | --- | --- | --- |
| *Rank* | *Models* | *Df* | *logLik* | *AICc* | *Δ AICc* | *AICc weights* |  | *Rank* | *Models* | *Df* | *logLik* | *AICc* | *Δ AICc* | *AICc weights* |
| **1** | **2/3** | **6** | **-378.495** | **769.14** | **0** | **0.222** |  | **1** | **1/2/3/4** | **8** | **-160.597** | **337.961** | **0** | **0.285** |
| **2** | **2/3/6** | **7** | **-377.918** | **770.035** | **0.895** | **0.142** |  | **2** | **1/2/3/4/6** | **9** | **-160.009** | **338.98** | **1.019** | **0.171** |
| **3** | **2** | **5** | **-380.231** | **770.568** | **1.428** | **0.109** |  | **3** | **1/2/3/4/5** | **9** | **-160.475** | **339.912** | **1.951** | **0.108** |
| **4** | **1/2/3** | **7** | **-378.423** | **771.045** | **1.906** | **0.086** |  | 4 | 1/2/3/4/5/6 | 10 | -159.892 | 340.966 | 3.005 | 0.064 |
| 5 | 1/2/3/4 | 8 | -377.574 | 771.405 | 2.265 | 0.072 |  | 5 | 1/3 | 6 | -164.403 | 341.248 | 3.288 | 0.055 |
| 6 | 1/2/3/6 | 8 | -377.845 | 771.947 | 2.807 | 0.055 |  | 6 | 1/2/3 | 7 | -163.547 | 341.686 | 3.726 | 0.044 |
| 7 | 1/2/3/4/6 | 9 | -376.942 | 772.206 | 3.066 | 0.048 |  | 7 | 1/2/4 | 7 | -163.578 | 341.749 | 3.788 | 0.043 |
| 8 | 1/2 | 6 | -380.142 | 772.433 | 3.293 | 0.043 |  | 8 | 1/2/3/6 | 8 | -162.625 | 342.016 | 4.056 | 0.038 |
| 9 | 1/2/3/5 | 8 | -378.171 | 772.599 | 3.459 | 0.039 |  | 9 | 1/2/3/4/5/6/7 | 11 | -159.3 | 342.028 | 4.067 | 0.037 |
| 10 | 3 | 5 | -381.251 | 772.608 | 3.468 | 0.039 |  | 10 | 1/3/5 | 7 | -163.853 | 342.299 | 4.338 | 0.033 |
| 11 | 1/2/4 | 7 | -379.308 | 772.815 | 3.675 | 0.035 |  | 11 | 1/2/3/5 | 8 | -163.048 | 342.861 | 4.901 | 0.025 |
| 12 | 1/2/3/4/5 | 9 | -377.475 | 773.272 | 4.132 | 0.028 |  | 12 | 3 | 5 | -166.324 | 342.962 | 5.001 | 0.023 |
| 13 | 1/2/3/5/6 | 9 | -377.595 | 773.511 | 4.371 | 0.025 |  | 13 | 1/2/3/5/6 | 9 | -162.172 | 343.307 | 5.346 | 0.02 |
| 14 | 1/2/3/4/5/6 | 10 | -376.848 | 774.09 | 4.95 | 0.019 |  | 14 | 2/3 | 6 | -165.495 | 343.432 | 5.471 | 0.019 |
| 15 | 1/3 | 6 | -381.178 | 774.505 | 5.365 | 0.015 |  | 15 | 2/3/6 | 7 | -164.595 | 343.782 | 5.821 | 0.016 |
| 16 | 1/3/5 | 7 | -380.94 | 776.078 | 6.938 | 0.007 |  | 16 | 1/2 | 6 | -166.346 | 345.135 | 7.174 | 0.008 |
| 17 | 1/2/3/4/5/6/7 | 11 | -376.825 | 776.122 | 6.982 | 0.007 |  | 17 | 2 | 5 | -167.867 | 346.048 | 8.087 | 0.005 |
| 18 | (Null) | 4 | -384.085 | 776.241 | 7.101 | 0.006 |  | 18 | 1 | 5 | -168.02 | 346.354 | 8.393 | 0.004 |
| 19 | 1 | 5 | -383.989 | 778.085 | 8.945 | 0.003 |  | 19 | (Null) | 4 | -169.454 | 347.116 | 9.155 | 0.003 |

Supplementary Table 11. Maximum likelihood parameter estimates and their standard errors of the top-ranked non-segmented linear mixed effect models for the **Ө**  treatment in the first (1993-2010) and second (2005-2018) data series. Temperature is here represented by mean summer temperature (°C). Level of significance: *, ≤ 0.05; **, ≤ 0.01; ***, ≤ 0.001.

| 1993 – 2010 | (Intercept) | Year | Elevation | Temperature | Elevation × Temperature |  |  |  |
| --- | --- | --- | --- | --- | --- | --- | --- | --- |
| 1 | -88.917 ± 18.634 *** | 0.044 ± 0.009 *** |  |  |  |  |  |  |
| 2 | -90.593 ± 18.697 *** | 0.045 ± 0.009 *** | -0.007 ± 0.003 * | -0.048 ± 0.027 | 0.001 ± 0 * |  |  |  |
| 3 | -89.071 ± 18.642 *** | 0.044 ± 0.009 *** | -0.002 ± 0.002 |  |  |  |  |  |
| 4 | -89.528 ± 18.714 *** | 0.045 ± 0.009 *** |  | -0.005 ± 0.017 |  |  |  |  |
|  | Variable importance: | 1.000 | 0.416 | 0.370 | 0.210 |  |  |  |
|  |  |  |  |  |  |  |  |  |
| 2005 – 2018 | (Intercept) | Temperature | Year | Elevation | Temperature × Year | Elevation × Temperature | Elevation × Year | Temperature × Year × Elevation |
| 1 | 166.664 ± 129.139 | -24.04 ± 13.887 | -0.083 ± 0.064 |  | 0.012 ± 0.007 |  |  |  |
| 2 | 156.584 ± 128.48 | -22.823 ± 13.806 | -0.078 ± 0.064 | 0.009 ± 0.008 | 0.011 ± 0.007 | -0.001 ± 0.001 |  |  |
| 3 | -1.179 ± 0.519 * | 0.126 ± 0.049 ** |  | 0.009 ± 0.008 |  | -0.001 ± 0.001 |  |  |
| 4 | -0.671 ± 0.295 * | 0.049 ± 0.028 |  |  |  |  |  |  |
| 5 | -47.602 ± 36.863 | 0.042 ± 0.028 | 0.023 ± 0.018 |  |  |  |  |  |
| 6 | -47.586 ± 36.926 | 0.119 ± 0.049 * | 0.023 ± 0.018 | 0.01 ± 0.008 |  | -0.001 ± 0.001 |  |  |
| 7 | -57.228 ± 36.236 |  | 0.028 ± 0.018 |  |  |  |  |  |
| 8 | -0.242 ± 0.164 |  |  |  |  |  |  |  |
| 9 | 167.297 ± 129.152 | -24.03 ± 13.886 | -0.083 ± 0.064 | -0.003 ± 0.005 | 0.012 ± 0.007 |  |  |  |
| 10 | -146.55 ± 221.477 | 10.472 ± 23.8 | 0.072 ± 0.11 | 5.411 ± 3.224 | -0.005 ± 0.012 | -0.594 ± 0.345 | -0.003 ± 0.002 | 0 ± 0 |
| 11 | -0.5 ± 0.383 | 0.049 ± 0.028 |  | -0.003 ± 0.005 |  |  |  |  |
|  | Variable importance | 0.846 | 0.645 | 0.502 | 0.389 | 0.382 | 0.061 | 0.061 |

Supplementary Table 12. Maximum likelihood parameter estimates and their standard errors of the top-ranked non-segmented linear mixed effect models for the **ꓵ** treatment in the first (1993-2010) and second (2005-2018) data series. Temperature is here represented by mean summer temperature (°C). Level of significance: *, ≤ 0.05; **, ≤ 0.01; ***, ≤ 0.001.

| 1993 – 2010 | (Intercept) | Temperature | Year | Temperature × Year | Elevation |  |  |
| --- | --- | --- | --- | --- | --- | --- | --- |
| 1 | -28.465 ± 14.792 | 0.037 ± 0.016 * | 0.014 ± 0.007 |  |  |  |  |
| 2 | -87.143 ± 56.489 | 7.289 ± 6.736 | 0.043 ± 0.028 | -0.004 ± 0.003 |  |  |  |
| 3 | -0.691 ± 0.186 *** | 0.043 ± 0.015 ** |  |  |  |  |  |
| 4 | -28.314 ± 14.809 | 0.037 ± 0.016 * | 0.014 ± 0.007 |  | -0.002 ± 0.005 |  |  |
|  | Variable importance: | 1.000 | 0.805 | 0.254 | 0.153 |  |  |
| 2005 – 2018 | (Intercept) | Elevation | Temperature | Year | Elevation × Temperature | Temperature  × Year | Elevation × Year |
| 1 | -80.33 ± 32.708 * | -0.023 ± 0.007 ** | -0.074 ± 0.053 | 0.04 ± 0.016 * | 0.002 ± 0.001 * |  |  |
| 2 | 58.818 ± 134.446 | -0.022 ± 0.007 ** | -15.822 ± 14.747 | -0.029 ± 0.067 | 0.002 ± 0.001 * | 0.008 ± 0.007 |  |
| 3 | -49.893 ± 69.135 | -0.547 ± 1.046 | -0.069 ± 0.054 | 0.025 ± 0.034 | 0.002 ± 0.001 * |  | 0 ± 0.001 |
|  | Variable importance: | 1.000 | 1.000 | 1.000 | 1.000 | 0.304 | 0.191 |

Supplementary figures


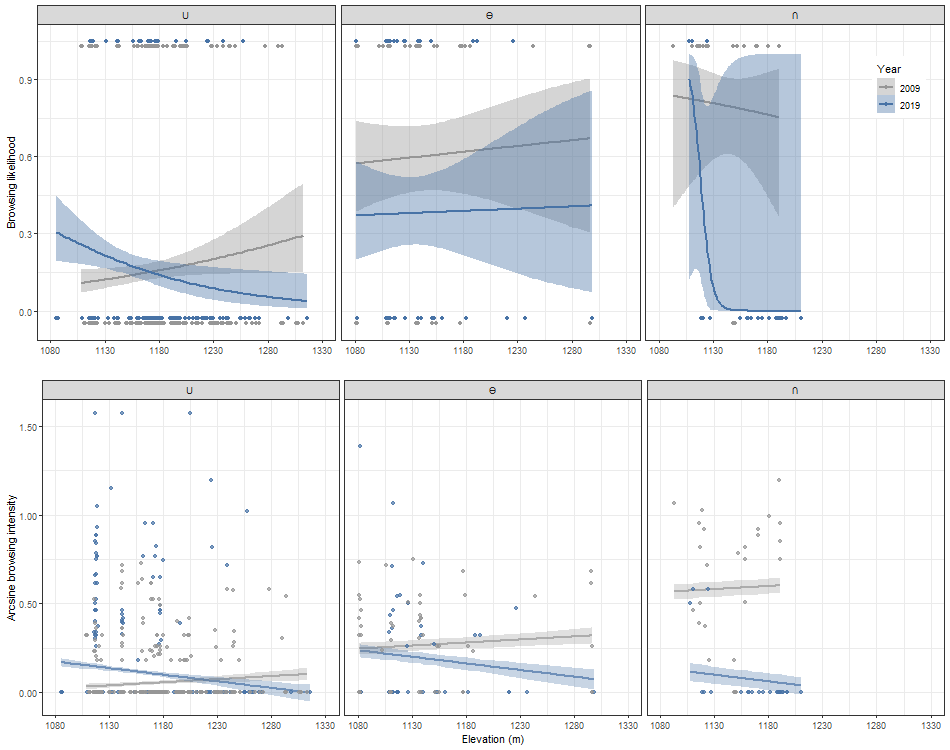


Supplementary Figure 1. Browsing likelihood as predicted by logistic regression and browsing intensity as predicted by linear regression in relation to elevation per treatment (**ꓴ,** **Ө**, **ꓵ**) per year (2009, 2019) for birch trees < 175 cm in height and < 30 mm in basal stem diameter. Dotted points at the top and the bottom of the browsing likelihood panels represent browsed and unbrowsed birch trees along the elevational gradient for each year, respectively.
